# Connecting Free Improvisation Performance and Drumming Gestures Through Digital Wearables

**DOI:** 10.3389/fpsyg.2021.576810

**Published:** 2021-04-12

**Authors:** Amandine Pras, Mailis G. Rodrigues, Victoria Grupp, Marcelo M. Wanderley

**Affiliations:** ^1^Digital Audio Arts, Department of Music, University of Lethbridge, Lethbridge, AB, Canada; ^2^Centre for Interdisciplinary Research in Music Media and Technology (CIRMMT), McGill University, Montreal, QC, Canada; ^3^Digital Arts, College of Arts and Sciences, Stetson University, DeLand, FL, United States

**Keywords:** digital music instrument, wearable MIDI interface, tactile fabric design, free improvisation, musical gestures, drums performance, electronic music, practice-based research

## Abstract

High-level improvising musicians master idiosyncratic gesture vocabularies that allow them to express themselves in unique ways. The full use of such vocabularies is nevertheless challenged when improvisers incorporate electronics in their performances. To control electronic sounds and effects, they typically use commercial interfaces whose physicality is likely to limit their freedom of movement. Based on Jim Black's descriptions of his ideal digital musical instrument, embodied improvisation gestures, and stage performance constraints, we develop the concept of a modular wearable MIDI interface to closely meet the needs of professional improvisers, rather than proposing a new generic instrument that would require substantial practice to adapt improvisational techniques already acquired. Our research draws upon different bodies of knowledge, from theoretical principles on collaboration and embodiment to wearable interface design, in order to create a digital vest called *Track It, Zip It (TIZI)* that features two innovative on-body sensors. Allowing for sound control, these sensors are seamlessly integrated with Black's improvisational gesture vocabulary. We then detail the design process of three TIZI prototypes structured by the outcomes of a performance test with Black, a public performance by a novice improviser during the 2017 International Guthman Musical Instrument Competition, and measurements of sensor responses. After commenting on the strengths and weaknesses of the final TIZI prototype, we discuss how our interdisciplinary and collective process involving a world-class improviser at the very center of the design process can provide recommendations to designers who wish to create interfaces better adapted to high-level performers. Finally, we present our goals for the future creation of a wireless version of the vest for a female body based on Diana Policarpo's artistic vision.

## Introduction

This paper presents the case study of a collaboration project between a team of designers, a music producer who conducts research in improvisation studies, and professional musicians, to create a modular concept of wearable interfaces that control musical parameters in drumming with electronics. The project originated when Pras noticed the discomfort of world-class drummer and improviser Jim Black, who moved his electronic device back and forth from a table, a floor tom, and his lap to control electronic sounds while performing for a recording session^1^. With the intent to find a more ergonomic way for Black to perform simultaneously on acoustic drums and electronics, Pras proposed the design of a wearable digital music instrument.

Digital musical instruments (DMIs) consist of gestural interfaces used to control sound synthesis parameters, with mapping strategies designed to connect performers' gestures to sound control variables. DMIs can take several forms, from emulations of acoustic musical instruments to totally novel devices that do not resemble existing instruments (Miranda and Wanderley, 2006). Performance with DMIs differs from performing on acoustic instruments as performer gestures do not have a direct physical correlation with the sound that is produced, i.e., one interface can control any number of sounds, sound libraries, or effect patches. Therefore, although DMIs allow for unlimited creativity, they necessitate clear artistic goals and performance metaphors for use in artistic contexts. Despite the creation of a growing number of DMIs in academic laboratories in the past decades, only a few became commercially available and captured musicians' attention outside of the academic sphere (Jordá, 2004; Sullivan et al., 2020). Indeed, most DMIs are designed to meet the idiosyncratic needs of a composer or performer who develops the device, often for an individual performer's use in a specific composition (Malloch and Wanderley, 2007; Rocha and Stewart, 2007; McPherson and Kim, 2012). In addition to favoring modular solutions that serve more than one artistic vision, we propose to create the space for musicians and researchers to collaborate throughout the construction of gestural interfaces. To do so, we follow the example of previous research in the music field (Pestova et al., 2009; Bacot and Féron, 2016; Martin, 2017) where professional musicians are not just passive users of an already terminated project, but their practice is at the center of the design process.

We apply a model of practice-based research that was developed by Edmonds and Candy (2010) in the context of interactive digital arts. In this model, the practitioner follows a trajectory that cyclically articulates three elements, e.g., Practice, Theory and Evaluation, to generate outcomes that should be “accessible to other people and therefore be available in a documented public form.” In our case study, the practitioner is represented by the team of DMI designers. In the model, the outcomes of Practice are Works, i.e., the DMI prototypes in our case study. The authors stress the importance of “experiencing these works […] for a full understanding of the practitioner's contribution to new knowledge.” Thus, in the discussion section, we report on the presentation of our final DMI prototype on stage at an official event, namely the 2017 Guthman Digital Musical Instrument Competition. In the model, the outcomes of Theory are Criteria and Framework described as “a living entity that can guide making, evaluating and interpreting.” In our case, the Criteria are the world-class improviser's explicit goals and artistic intentions that we collect through an interview, and the Framework is the ultimate design process to meet these goals and intentions. Moreover, the authors explain that Theory is also “likely to consist of different ways of examining, critiquing and applying areas of knowledge considered relevant to the individual's practice.” In this view, we provide a literature review of previous research that informs the artistic context of our project and that inspired our pre-established design approach. In the model, the outcomes of Evaluation are Results that are assessed according to the Criteria and that determine the Framework. In our case study, Results are an initial performance test and measurements of the prototypes' sensor responses.

We introduce the background of this case study with Mazzola and Cherlin's (2008) “Dimension of Embodiment” ontology and “Art of Collaboration” principles to establish the research context of free improvised performance. We then discuss the benefits of performers' re-appropriation of an everyday object according to the “Enacting Knowledge” theory (Bordegoni, 2011). We also situate the organizational framework of our design within the growing fields of soft computation with tactile fabric (Berzowska and Bromley, 2007). Methods and results sections present our trajectory to design three prototypes of a digital wearable named *Track It, Zip It* (TIZI) that aim to facilitate Black's performance. In the conclusion, we highlight the most recent stages of the project that involve visual and sound artist Diana Policarpo and the development of an extended and wireless version of the improved TIZI prototype for a female body. While the technological insights that we include in this paper would enable the replication of our wearable DMI, our main purpose is to contribute to new modular designs with the concept of our design trajectory in order to create gestural interfaces that meet the requirements of high-level musicians.

## Literature Review

### The Dimension of Embodiment in Free Jazz/Free Improvisation

Our case study takes place in a specific music performance practice commonly known as free jazz, though referred to as free improvisation^2^ by the musicians who participated in the project (Pras et al., 2017). Mazzola and Cherlin (2016, p. 33–34) introduced the “Dimension of Embodiment” as an extension of previous ontologies to study music. This dimension includes three values that deal with respective activities: (1) Gestures with “to make,” (2) Process with “to refer to,” and (3) Facts with “to be the case.” While the need to distinguish between improvising processes and improvisatory outcomes (facts) have been discussed in academic literature about improvisation (e.g., Arthurs, 2016; Pras, 2016; Pras et al., 2017), the gestural value has yet received scant attention. This contrasts with the DMI field, where researchers have always emphasized gesture definitions to engineer innovative correlations between musical gestures and sound production (e.g., Cadoz and Wanderley, 2000).

Considering that free jazz, or free improvisation, can be distinguished from other music practices by the absence of pre-canvas while making music, Mazzola and Cherlin (2016, p. 65) define gesture as “the technical tool of communication and creative flow, but it is also the point of no return for the transition from the world of facticity to the world of the making.” In this music practice, gestures represent the artists' technical tools of pitch, time, textures, intensity, and position to compose music instantaneously. Previous work within the free improvisation scene of New York City showed that improvisers' creative and thought processes strongly varied from one artist to another, and that shared understanding about these processes is not necessary to improvise together successfully (Pras et al., 2017). Our project thus leaves the mappings and sound design to the improvisers who need to maintain control of their idiosyncratic processes. We focus instead on the gestural interface, and we ground our pre-established design approach in the acquired gestures/technical tools of free improvisers, rather than proposing or imposing new gestures/tools that would automatically limit these performers' ability to make music.

Previous research showed that improvisers who perform simultaneously on acoustic instruments and electronic devices are likely to bring different means to their use of electronic gestures. Indeed, a previous investigation of Black's and Satoshi Takeishi's electronic gestures in solo improvisation highlighted two different approaches^3^. While Black emphasized his need to extend the sonic possibilities of a drum set with an interface that would give him access to melodic and harmonic expression, Takeishi discussed the aesthetic challenges of performing with electronic controllers for an audience (Lavergne and Pras, 2014; Pras, 2016). In response, we are involving a second high-level drummer for the next step of our research, Diana Policarpo, who wishes to extend Black's gestural needs with visual aesthetic requirements.

Free jazz improvisation communities share common ideation principles that Mazzola and Cherlin (2016, p. 29–37) conceptualized as the “Art of Collaboration.” For this project, we involved music performers at the center of the design process and established a collaborative workspace that allowed for “gesture-based collaboration,” aware that “differences in language, style of thinking, and understanding of the very concept of a discipline are too strong for an unprepared exchange of knowledge, technology and inspiration.” We thus created an environment of mutual understanding by fostering exchanges with professionals who were open to sharing their experience and knowledge about designing DMIs or performing with DMIs. These exchanges took place in physical workspaces, thus were not limited by web communication constraints—enabling us to learn well beyond technical advice.

### Designing a Modular DMI as an Everyday Object for Performers' Re-Appropriation

Bordegoni (2011, p. 75–76) defines “Enactive Knowledge” as “the information that the user gains through perception-action interaction in the environment (…). Typically, enactive knowledge is related to manual tasks—humans' own knowledge on concrete actions in their hands, which are used for grasping, manipulating, handling, and shaping.” These manual tasks include various activities that require fine dexterity such as knitting, pottery-making, or playing an acoustic music instrument. In this view, the success of Moog keyboards in the 1960s in jazz and pop music is attributed to the introduction of an enactive knowledge of piano-playing in the control of analog synthesizers (Fantinatto, 2014).

Previous research in Music Technology shows that a balance between the moderate ease of play and the feeling of control increases performers' interest to adopt a DMI (Cook, 2003; Ghamsari et al., 2013). Likewise, a well-known object shape implies an ensemble of gestures that help performers to explore a new DMI in a way that is directed by the device itself (Rocha and Stewart, 2007). For example, the ball shape of “Intonaspacio” suggests the enactive knowledge of idiomatic actions such as throwing, rolling, passing to others, etc. (Mamedes et al., 2014; Rodrigues et al., 2014). Similarly, DMIs that are designed for percussionists usually feature a flat surface (Lai, 2009; Bin et al., 2017; Martin, 2017). Such designs enable performers to quickly feel in control when using the devices. With the pre-established goal of designing a wearable interface to facilitate simultaneous playing on drums and electronics, we gave drummer Jim Black a choice between two everyday pieces of clothing: a shirt or a vest. Consequently, he and other drummers would not need to learn how to manipulate the interface from scratch. Instead, they would be able to re-appropriate the enactive knowledge of wearing a shirt or a vest into a musical instrument for their performance needs. This advantage is crucial when designing a DMI according to the needs of touring artists whose professional schedules do not allow for the long-term learning of a new instrument.

### Parallels Between the “Art of Collaboration,” the Maker Movement, and Soft Computation

The emergence of inexpensive and plug-and-play electronic sensors and microcontrollers such as the Arduino^4^ has facilitated the emergence of makers' internet communities around sharing tips, instructions, and basic knowledge on electronics, allowing non-engineers to build electronic projects and express their creativity through digital design. This recent democratization of digital design influences DMI communities as well, with several composers and performers developing their own devices without depending on engineers or programmers, though sometimes with limited capabilities. For instance, many musicians' DMIs are introduced at the NIME conference^5^ every year. Results from a study of NIME interfaces between 2009 and 2013 (Medeiros and Wanderley, 2014) reveal that most DMIs “are based on a few basic sensors types and unsophisticated engineering solutions, not taking advantage of more advanced sensing, instrumentation and signal processing techniques that could dramatically improve their response.” There is also, in general, limited use of textile sensors, as designers tend to focus only on a handful of technologies (Medeiros and Wanderley, 2014). As a consequence, we observe very similar gestures from one interface to another, which strongly reduces the expressive capability of these devices and their eventual adoption by accomplished improvisers. The multidisciplinary expertise of our team enables us to incorporate the creative and positive social aspects of the maker movement with engineering, and to bridge the gap between academic engineering and a non-academic music performance context.

We found valuable inspiration in the literature on soft computation, a field that is contemporary to affordable electronics. Soft computation was defined by Berzowska and Bromley (2007) as “the design of electronic technology that is composed of soft materials such as textiles and threads. Soft computation is also predicated on traditional textile construction methods such as sewing, embroidery, and appliqué [sic] with various conductive and active materials to create interactive fabrics” (Berzowska and Bromley, 2007). Currently, wearables are extensively developed for medical (Patel et al., 2012), fashion (Seymoun and Bello, 2008), dance performance (Sicchio et al., 2016), and interactive installations (Giordano et al., 2015; Lamontagne et al., 2015). As musical interfaces, wearables have been predominantly materialized in the form of gloves (Sonami, 2014), and bodysuits (Bhagwati, 2018).

## Research Questions and Objectives

Our main goal is to reflect upon the involvement of a world-class improviser at the center of the design process of a wearable gestural interface that aims to extend the performers' body and facilitate creative gestures. To do so, we will answer the following research questions:

RQ1: To what extent can our design approach for a custom-made wearable meet a world-class improviser's criteria for an ideal DMI in the context of solo performances on acoustic drums and electronics?
RQ2: Does the embodiment of a fabric-based wearable DMI, whose access is immediate and intimate, enhance or limit improvisation gestures in music-making?
RQ3: To what extent can a wearable modular interface that is custom-made for a specific world-class improviser be adopted by a novice improviser?

## Methods

### Approach

The methods of our practitioner trajectory (Edmonds and Candy, 2010) are inspired by the cognitive anthropology approach of Donin and Theureau (2007) who examined, in depth, the creative process of music composition with acoustic instruments and electronics. This cognitive anthropology is appropriate for conducting case studies in real-life settings as it invites researchers to collect a set of data that informs the different aspects of the creative process, including interviews of the composers, performers, and other agents involved in the creation (e.g., audio engineers); screen captures of music composition software; photos of the scores at different stages of the composition; video recordings of the rehearsals; and email exchanges between the different agents of the creation. Donin and Theureau introduced this methodology for the study of the creative process of *Voi(Rex)* composed by Leroux (2010). After the study, Leroux reflected upon the impact of the research process on his compositional process (2010). The methodology was then applied to study the creative process of other compositions, e.g., the beginning of *Gramigna* by Stefano Gervasoni (Donin and Féron, 2012). For our case study, we adapted the data collection methods of this cognitive anthropology to inform the design trajectory of our wearable DMI.

DMI scholarship has also developed methodologies that incorporate different types of data collections to inform the design trajectory. For instance, Garcia et al. (2014) combined digital captures and feedback questionnaires to test *Polyphony*, a novel interface that “integrates interactive paper and electronic user interfaces for composing music.” While this study has the benefit of allowing several composers to test the novel device, the evaluation is restricted to a controlled composition task. For our study, we wanted our design to meet the requirements of high-level improvisers in real-life settings. We thus chose to detail the design process of a digital vest named *Track It, Zip IT* (TIZI) according to the wishes of world-class improviser Jim Black. For future research, we wish to explore the potential of extending this wearable interface to other types of bodies and means of expression. We therefore invited the visual and sound artist Diana Policarpo to collaborate with us in order to create an extended version of *TIZI* for female drummers that would also include new features that correspond to her artistic vision.

### Design Trajectory of Three TIZI Prototypes

#### Interview With Jim Black

Considered one of the best drummers of our time by music critics, Jim Black started his international performer's career at the age of 17. Originally from Seattle, based in New York between 1991 and 2016, and now based in Berlin, he has toured around the world with his bands of alternative jazz and has drummed for bandleaders as diverse as Laurie Anderson, Steve Coleman, or Dave Liebman. He started using technology such as effect pedals and amplification to create and manipulate sound in 1984. Since 1999, he has enhanced his drum kit with electronic sound sampling and looping for album productions, e.g., his album *Malamute* (Black, 2017). Black is currently using the modular ROLI Seabord Block and endorses the company.

Grupp interviewed Black under Pras' mentorship for about an hour at Stetson University's sound studio on February 12, 2016. Digital artist Matt Roberts, another of Grupp's mentors, was also present during the interview and helped ensure that the technical discussions led to feasible design goals. A video recording of this interview shows a medium distance shot of Black and captures his explanatory gestures and comparisons with the ROLI Seaboard RISE 25, which he had used in performance the previous evening^6^. The interview guide included several semi-directed questions encouraging Black to express his main wishes and requests for the wearable interface that Grupp at first referred to as a shirt. To keep the list of design requirements manageable for a short design timeframe, she asked him to name five main things that he would like this shirt to do. She then gave him a choice between a vest or a shirt, with the possibility of using one of his own. After making sure that Black did not want us to be involved in the mapping and sound design of the interface, we discussed the number of MIDI channels that would be made available. Finally, we brainstormed design ideas and sensor placements, which led Black to simulate a few gestures on his body.

We extracted design criteria from the analysis of Black's list of requirements, his gestural demonstrations, and our technical brainstorming during the interview. With these design criteria, Grupp applied for student funding under the supervision of Pras to realize this project.

#### Design Approach for the Initial TIZI Prototype

Grupp and Rodrigues designed the first prototype of *Track It, Zip It* (TIZI) at IDMIL between May and June 2016, based on the criteria that we had identified from the analysis of Black's interview. The creative process included weekly brainstorming sessions with the team, and exchanges with external experts such as Rodolphe Koehly^7^, who assisted in the design of low-cost custom electronic sensors. Throughout the design process, we also consulted with trombonist Felix Del Tredici about his experience wearing a bodysuit (Bhagwati, 2018) to perform pieces by composer Sandeep Bhagwati^8^.

#### Performance Test With Jim Black

We organized a test session at the Immersive Presence Lab at CIRMMT with Black on June 27, 2016. Black was touring with his band *Endangered Blood*. They had a concert in Ottawa the night before and a another one in Montreal that night. Consequently, we had the opportunity to book him for a whole afternoon to test the vest. A few days before the test, Grupp sent him a MaxMSP patch to ensure that the first TIZI prototype could interface with his Reaktor patches and sound libraries. The technical setup for the test included a drum kit^9^, two speakers to play back the electronics, lighting, a couple of cameras, and audio recording equipment to document the performances (see Supplementary Material). We invited Del Tredici to improvise with Black during the test, which resulted in a 12-min duo performance using TIZI, followed by a solo experimentation of the sensors.

#### Design Approach for the Improved TIZI Prototypes

Grupp and Rodrigues designed the second prototype of TIZI during the last week of Grupp's residency at IDMIL between June 28 and July 8, 2016. Based on Black's feedback and on our gesture analysis of the test performance video, Grupp and Rodrigues modified the first prototype and fixed issues while recycling the electronic material of the initial prototype. The second TIZI prototype is now part of IDMIL's repertoire of DMIs.

In July 2016, Grupp designed the third TIZI prototype back in Florida to fix sensor response flaws that Rodrigues identified in the second prototype measurements once she had left IDMIL. A video demo of this third prototype was submitted to the Guthman Musical Instrument Competition in November 2016 (see Supplementary Material). Grupp later presented this third prototype as a semi-finalist of this competition at Georgia Tech in Atlanta, GA, on March 9, 2017. The presentation included a 10-min talk about the design, and a demonstration with Blake Rook, a cello performance major from Stetson University who had a solid experience in drumming—though limited access to a drum kit during his studies. Rook had been writing and performing music for about 10 years when he started experimenting with the third TIZI prototype in the sound studio of Stetson University that featured a drum kit. Grupp and Rook collaborated on the mapping between gestures and sound effects through a trial-and-error process. Specifically, Grupp would present Rook two or three mapping options, and Rook would select the one that felt the best for improvising. They rehearsed like this for a couple of weeks prior to the competition for the purpose of demonstrating the device. In March 2017, this third prototype was sent to Black, who returned it to us in April 2020 in order for Rodrigues to perform some measurements of the sensors whose results are included in this paper.

## Results: Design Trajectory of TIZI Prototypes

**Figure 1:**
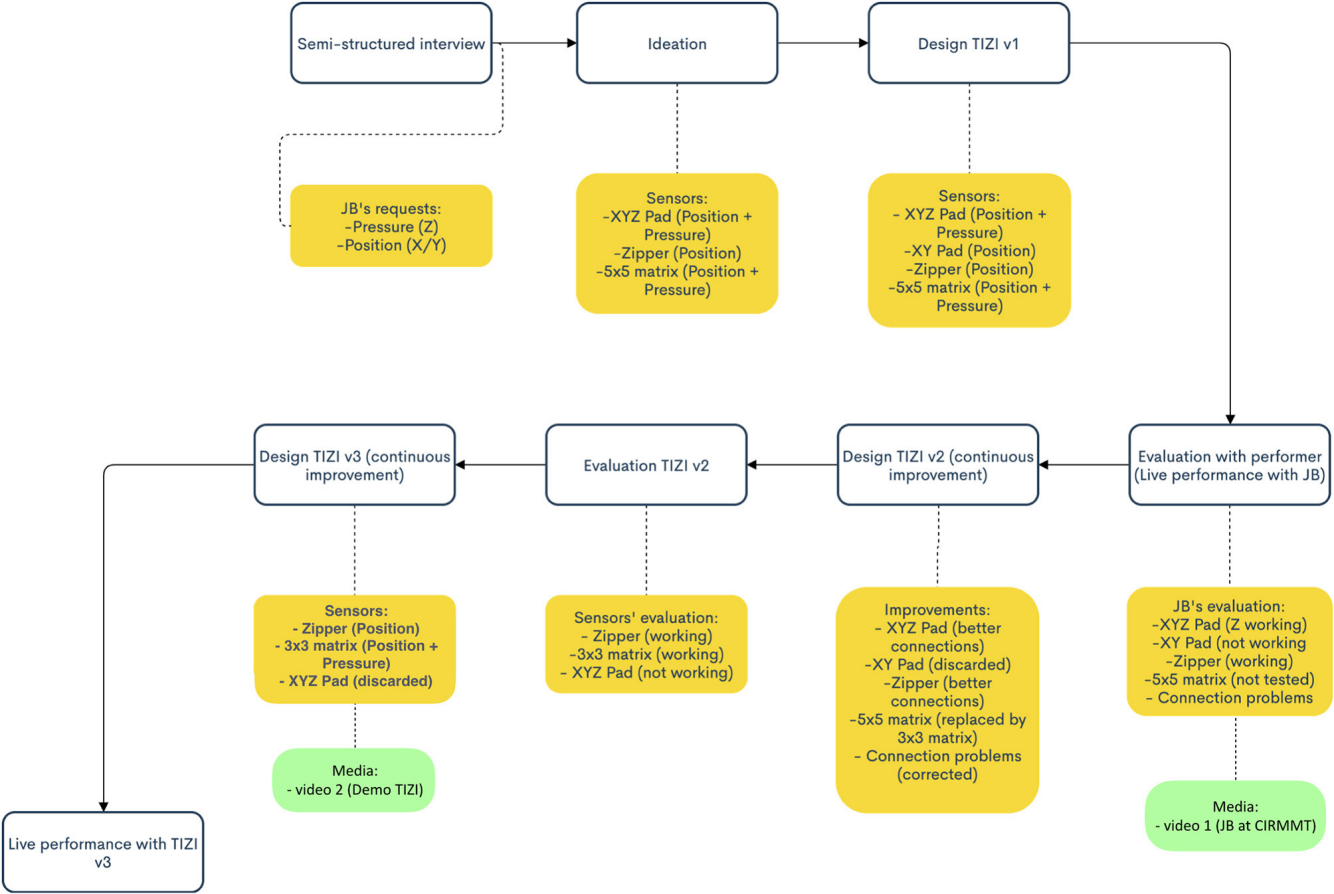
Flowchart of our design trajectory of the TIZI prototypes.

Figure 1 presents a flowchart of the design trajectory of three TIZI prototypes. This trajectory was structured in time by the performance test with Black that fit his touring schedule, by Grupp's grant-restricted IDMIL residency dates, and by the deadline for the Guthman Digital Music Instrument competition application.

### Criteria: Jim Black's Ideal Wearable DMI

When asked about the five main things that he would like the wearable interface to do, Black directly answered: “*I am not concerned about the controller, I am more concerned about what one can do with gestures*.” Then, he spontaneously said that “*the dream controller would be a surface with enough real-estate of XY-playing and Z-playing*” and that “*pressure is key*.” Later in the interview, he mentioned again that “*the dream piece would be a XYZ pad, I don't know what comes after XYZ with one gesture*.” As he put it: “*press and slide or press and move are musical gestures*.” His list also included triggers with pressure (single state buttons on/off and pressure sensors), and “*a knob or some sort of slider or control slip that would hold the function when you release the finger*.”

**Figure 2:**
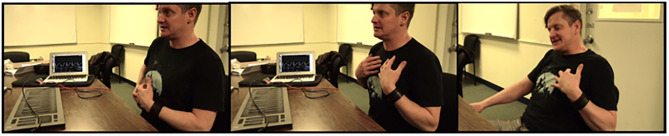
Snapshots from Black's interview when he demonstrated possible gestures on his torso.

Regarding the type of wearable, Black quickly chose a vest over a shirt because a vest would resist sweat better and require less washing when touring. He specified that he was not concerned about being too hot while performing as “*performance accuracy is the most important*.” As an example of a vest, he referred to an outfit to ride a motorcycle, Darth Vader's or Batman's costumes, or a “*superhero-vibe vest*.” Nevertheless, he agreed an armless vest would leave his arms and wrists entirely free to play the drums. This part of the interview included several gestural demonstrations on his torso (Figure 2).

Black mentioned that his position when playing the drums could be used to track wrist or leg motions, as well as back-and-forth torso movements. However, he expressed reservations regarding alternative MIDI devices that “*look great but don't do much*,” e.g., the MIDI glove or a pad that does not read pressure on the edges, while the ROLI allows him “*to move a lot of music around with a few gestures*.” In this view, he specified that “*the ROLI is tracking like a drum*,” which implied the requirement for a low latency device.

When asked about MIDI inputs, he answered that his Reaktor patch uses simple MIDI readings on five channels with values between 0 and 127 for attack, velocity, pressure, stereo balance, and release. He thus proposed that we generate two voices of these five readings that could be controlled by ten fingers. Later in the interview, he showed us one of his Reaktor patches and expressed wanting to send multitimbral data. This would require separating the MIDI channels into six distinct signal paths, enabling greater control and better latency than sending multitimbral data through a single MIDI channel.

### Work: Layout of the Initial Prototype

The development of the first *TIZI* prototype was divided into three rounds: (1) the concept and test of four individual fabric sensors: XY pad, XYZ pad, a linear potentiometer (using a zipper with metal teeth), and a 5 × 5 matrix; (2) the design of the vest; and (3) the assembly of the sensors on the vest. This process allowed flexibility in the final placement of these modular sensors on the garment.

**Figure 3:**
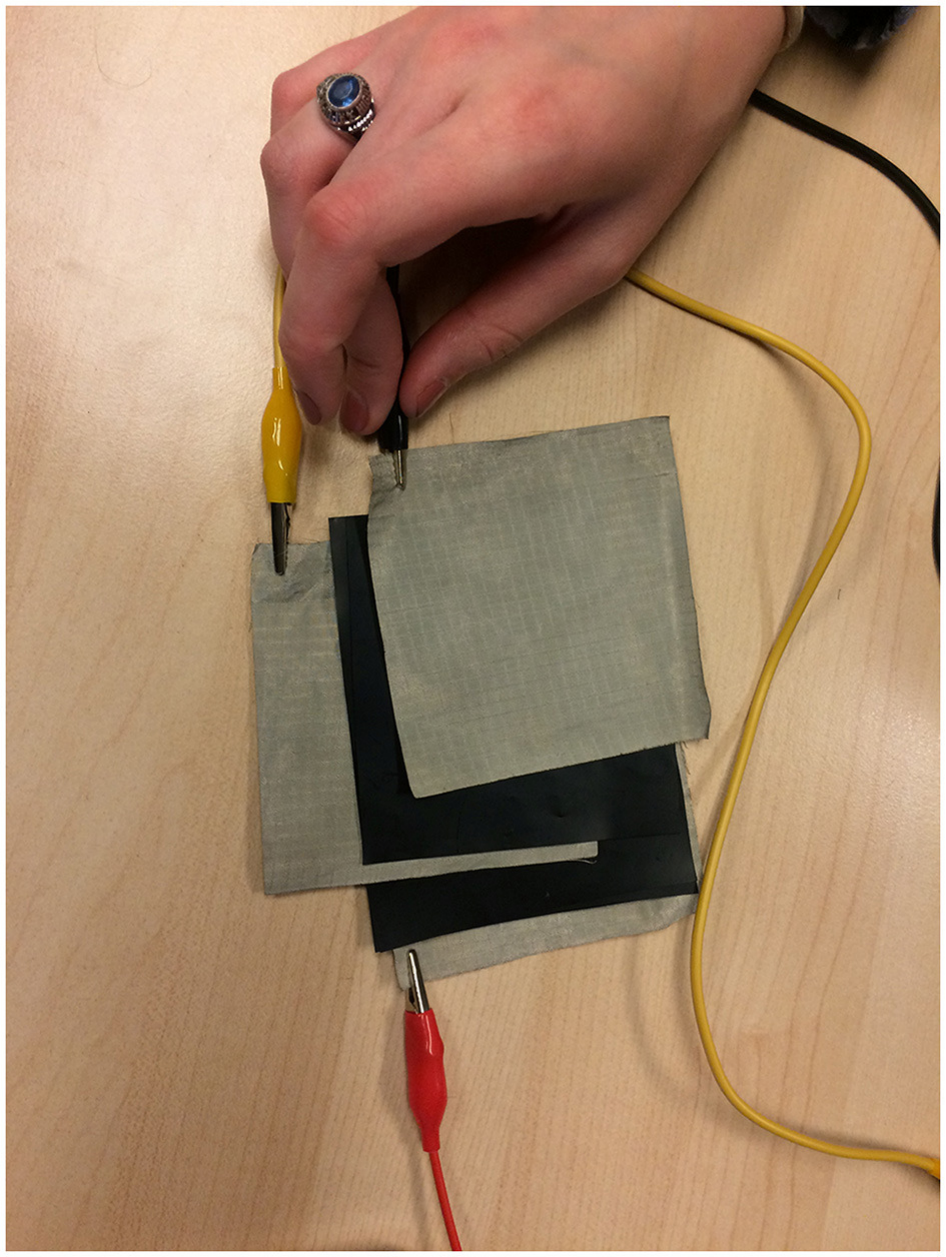
Six alternating layers of conductive fabric and Velostat to build a XYZ pad.

Following Black's primary desire for a pad that detected position and pressure, we dedicated a lot of time to designing a sensor that combined reading position (on an XY pad) using conductive fabric and reading resistance (to pressure or squeezing) using Velostat, an electrically conductive material^10^. This XY sensor has four intertwined layers: one layer of conductive fabric, followed by one layer of Velostat, followed by a second layer of conductive fabric and a second layer of Velostat. The Velostat generates a difference in resistance when the sensor is pressed. The sensor is fed voltage and ground, and readings are extracted in an orthogonal fashion in order to obtain positions on two axes (X and Y). Data on pressure (Z) is obtained using the same technique, but adding two extra layers, one of conductive fabric and one of Velostat, resulting in six layers in total (Figure 3).

We used a zipper with metal teeth to build a textile potentiometer. Because metal is a conductor, we sewed conductive thread at specific locations in the zipper to create steps of different readings once the zipper would be closed or opened, working as a voltage divider.

We also built a fabric 5 × 5 matrix from a paper matrix designed by Rodolphe Koehly. We used fabric instead of paper for sweatproof, washable, and robustness purposes. Koehly provided us with one of his matrix models and the algorithm that allowed us to explore the multi-touching functioning of his design. The matrix is composed of two layers of conductive fabric, separated by a layer of Velostat. In each layer of fabric, there are lines of conductive thread sewn in the fabric horizontally and vertically. These seams make the tactile as well as the visual structure of the matrix—creating rows and columns. Voltage is applied on one column at a time through the digital outputs of the microcontroller. Measurements are taken, column by column, in an iterative method. The moment one presses anywhere in the matrix, the change in resistance is detected.

To sketch the pattern of the vest shape, we removed the sleeves of a basic shirt. We chose a water-resistant fabric to protect the electronics from sweat. Two pockets filled with cardboard pieces were added to the vest to create a stable and stiff zone for the detection of pressure, and to ensure that the sensors would only detect actions when a user would actively press the sensitive zone.

For the electronic platform of our design, we selected Adafruit's Flora^11^, a plug-and-play microcontroller for wearable interfaces. Along with Sparkfun's LilyPad, Flora is the sewable microcontroller that offers the highest number of analog inputs (four) available on the market. Because we needed three analog inputs for the XYZ pad, two analog inputs for the XY pad, one analog input for the zipper, and four analog inputs for the 5 × 5 matrix, we sewed three Flora boards onto the back of the vest. Because this body region is the least affected by drumming movements, the board placement allowed the electronic connections to be secured and the device to output proper readings.

**Figure 4:**
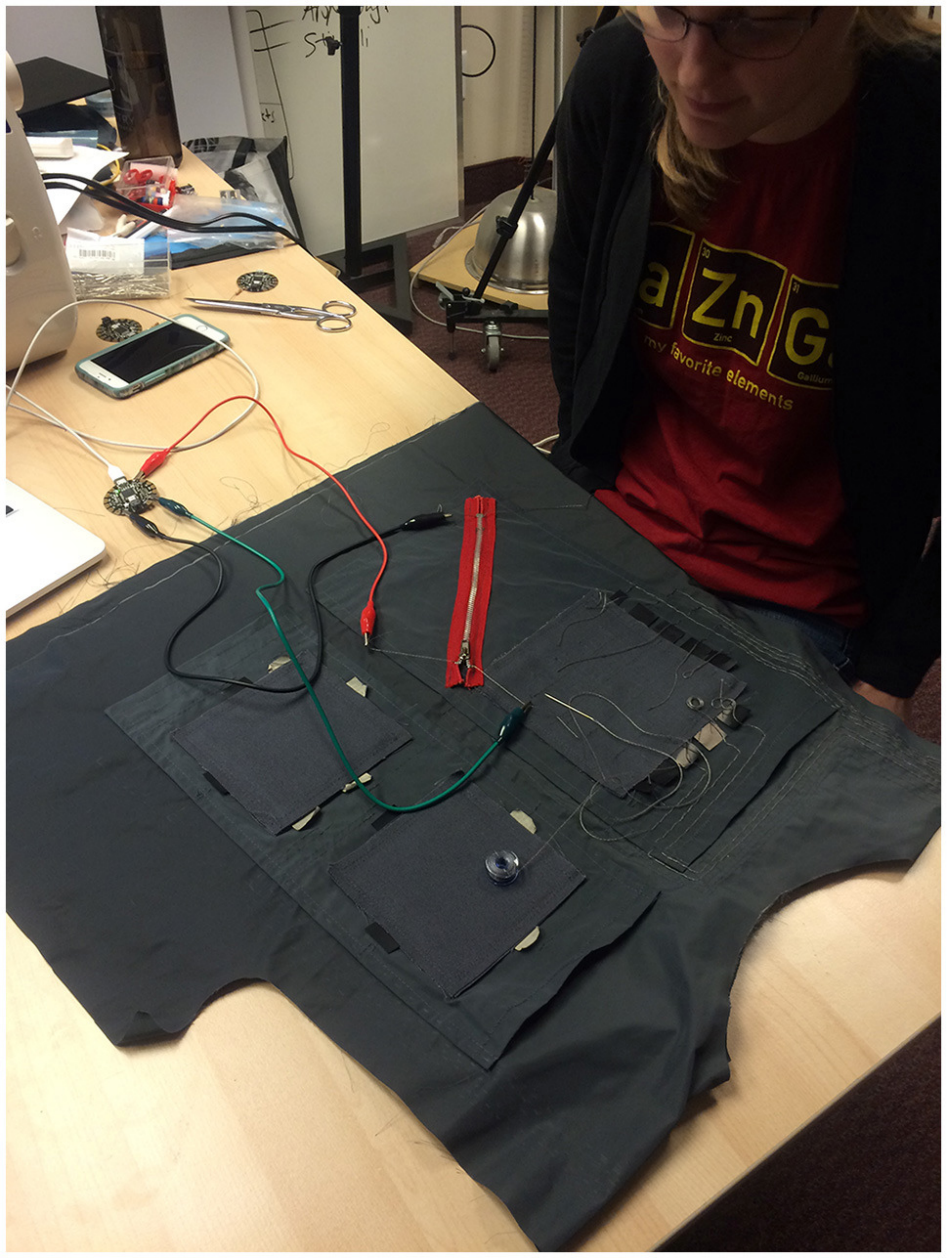
Placement of the sensors on the initial vest prototype (X/Y Pad, X/Y/Z Pad, zipper, and 5 × 5 matrix).

All four sensors were placed in the front of TIZI in strategic places that Black had indicated during his interview (Figure 4). Finally, the sensor outputs were mapped to MIDI values (0–127), according to what Black needed to receive in his Reaktor patches. The three Flora boards were connected to the computer using three micro USB-to-USB cables.

### Evaluation: Outcomes of the Performance Test With Jim Black

**Figure 5:**
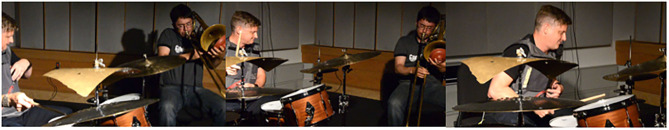
Snapshots of Black testing the initial *TIZI* prototype with Felix Del Tredici (trombone) at CIRMMT: he creates a cluster.

Figure 5 features snapshots from the video recording^12^ of Black's performance test of the first TIZI prototype at CIRMMT on June 27, 2016. At the time, only the XY pad, the XYZ pad, and the zipper were tested because the 5 × 5 matrix was not finished yet. Black individually mapped the three sensors as follows: both the XY and XYZ pads were programmed to modulate sound samples, using the position of his finger on the pad. The Z value (pressure) was assigned to trigger the sound. The zipper was mapped to a collection of sounds in a sound library: the position of the metal slider was associated with a given sound.

Several observations were made from the test of the three sensors on the initial prototype. First of all, the latency between Black's gestures and the sensors' response did not get in the way of the performance. Sound-triggering from the two pads and the zipper seemed to work as intended at the beginning of the performance. However, the modulation of sounds was quite difficult to perceive. The values detected on the X and Y axes of the two pads were very noisy—with the readings jumping from one value to another—which made the effect settings hard to control. We also noticed that the sensors were not sensitive enough to small gestures: when Black used more than one finger at a time to play the pads, small and fast movements would not affect the generated sound. This last constraint called for improvements in tracking detailed fingerings for more subtlety with the interface.

The cardboard pockets of the vest were uncomfortable for Black's playing, and the one on the left side continually moved out of its socket. During the test, we noticed that the pads started to behave randomly, for example triggering sounds when there was no touch or triggering sounds with a considerable delay. These technical issues were most likely due to torso movements that brought conductive thread lines placed on the back and shoulders of the vest to touch one other, and thus short-circuit.

**Figure 6:**
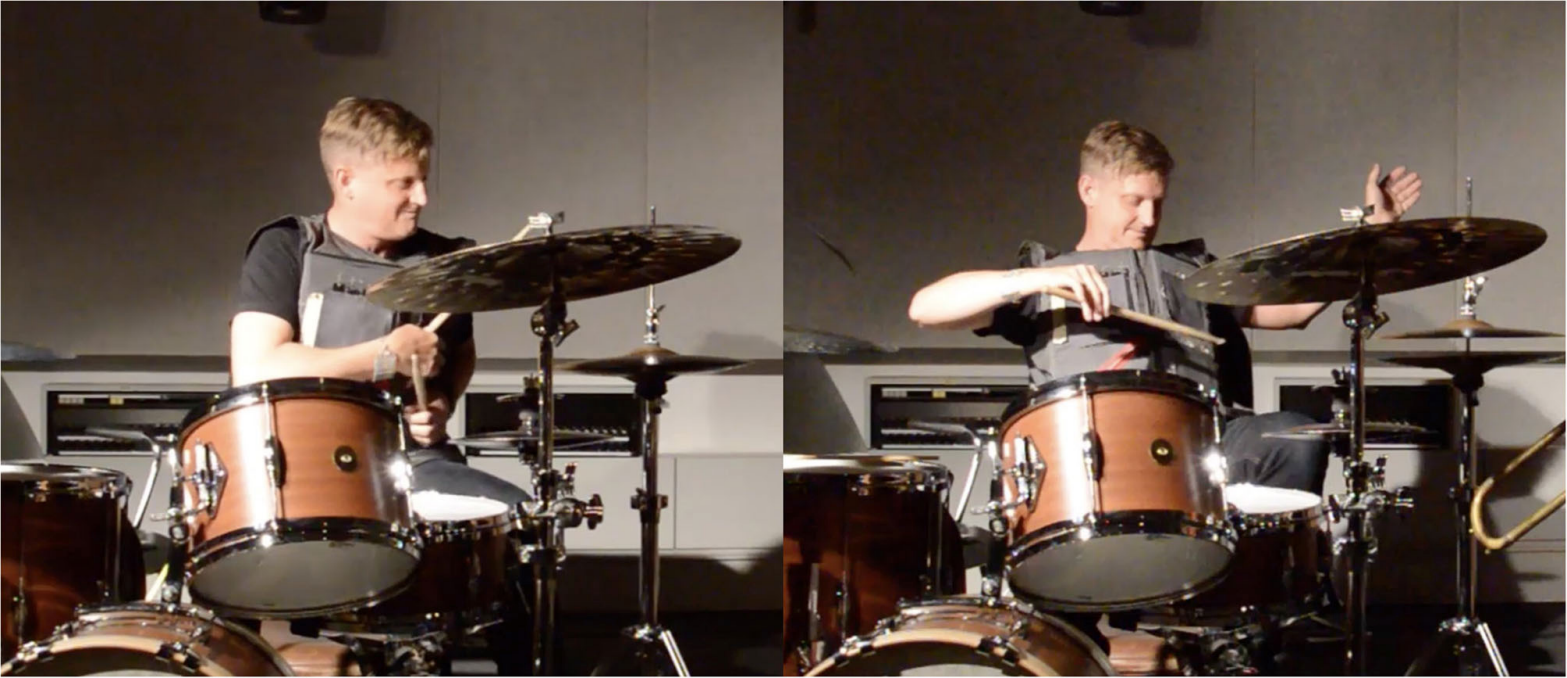
Black plays the vest using unexpected gestures: he uses his drumstick on the wearable.

Despite the issues that are described above, Black's movements looked natural, and the transition from the drums to the electronics, and vice-versa, occurred smoothly. He also seemed to be having fun when he could be in control of the sounds that were generated, e.g., “*the pressure thing, it does work, that's cool*.” Specific gestures captured our attention: at one point, he used his drumsticks instead of his fingers to activate the pads, whose visual effect was an effortless gesture that could enhance the flow of the performance. Another time, he created a cluster in the lower pad, which was an unexpected gesture (Figure 6). His invention of gestures on the spot while using the interface for the first time reflects the level of control that he could enjoy over the first TIZI prototype, thus corroborating the idea that this interface is straightforward to learn and to embody.

### Work: Design of the Second and Third Prototypes

**Figure 7:**
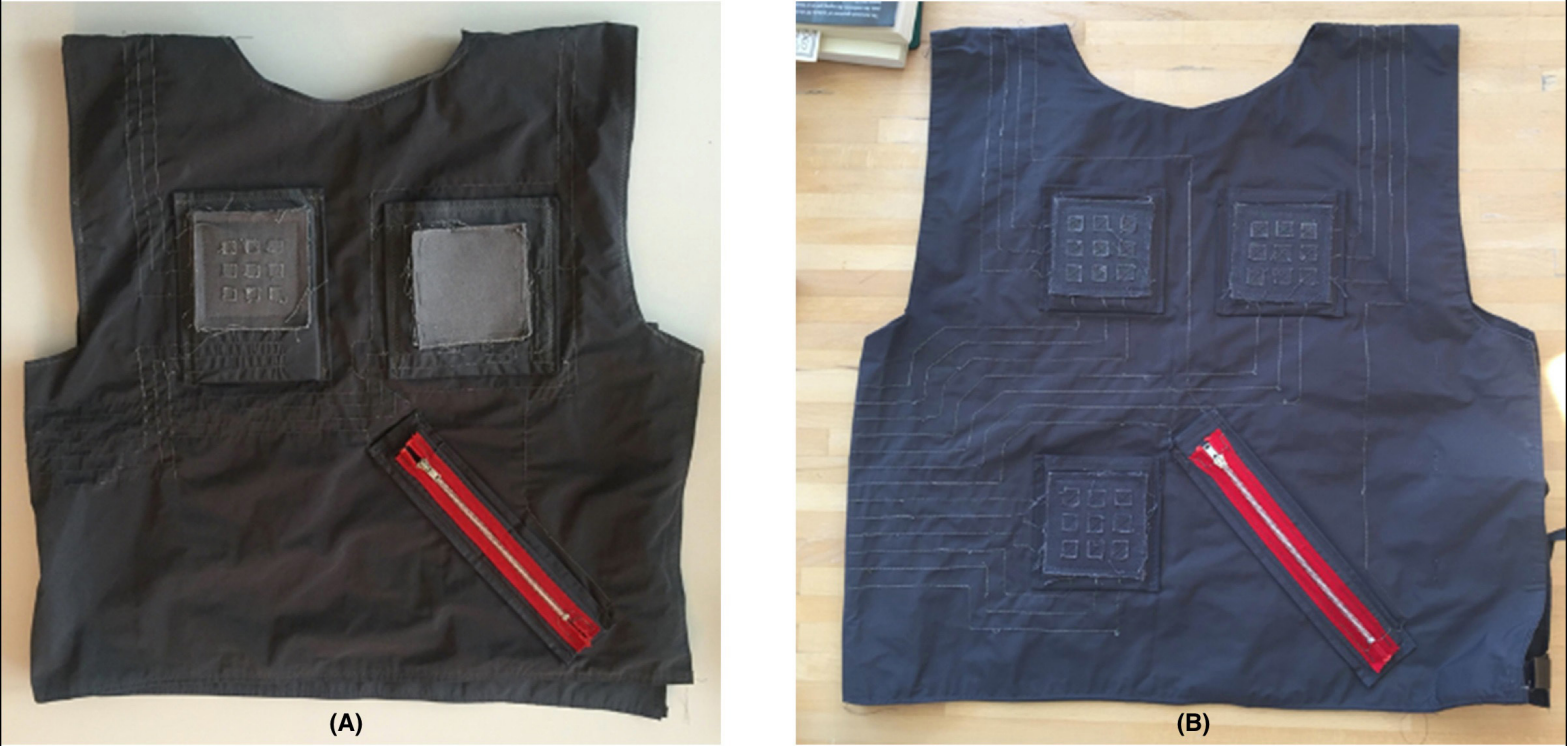
**(A)** Second version of TIZI; **(B)** Third version of TIZI.

The second version of TIZI addressed some of the technical issues observed during the test session at CIRMMT (Figure 7A). For instance, the second prototype did not include the XY pad that had not been convincing during the test but kept the XYZ pad because the Z (pressure) presented good potential. We reduced the original 5 × 5 matrix to a 3 × 3 matrix to reduce the number of digital outputs in the microcontroller. The stitches were completely isolated using nail polish to reduce the electric coupling. Furthermore, to avoid short-circuiting, the majority of stitches were passed under the armpits, leaving Voltage on one shoulder and Ground on the other shoulder. Hence, even if the fabric slid together in the shoulders due to upper-body movements, Voltage and Ground would not touch. The zipper remained the same, because it performed quite well during the entire test session.

The third TIZI prototype (Figure 7B) uses three 3 × 3 matrices and a zipper. This version thus combines the sensors that performed best in the previous version and that addressed Black's two most important requests: on/off and pressure (matrix) and holding position (zipper). The most important design decision was to discard the XYZ pad and only use matrices because we could not find a solution to get better reading accuracy for X and Y without using cardboard pockets or an equivalent stiff surface. In the third prototype, each of the three 3 × 3 matrices has larger buttons than on the second prototype to reduce the errors detected in the previous version. The goal was to provide drummers with a multitouch surface, as well as to account for more detailed tracking of the fingers tapping on the vest.

Due to the increase in the number of matrices, one extra Flora board was added to the third prototype. Three boards were dedicated to each of the three 3 × 3 matrices (three analog inputs), and the fourth one was connected to the zipper (one analog input). The choice of keeping the zipper on a separate microcontroller aimed to facilitate the control of each matrix individually. This gave us the possibility to add more sensors to the vest later. On the third prototype, we also used nail polish to isolate the stitches sewn to the teeth of the zipper. This small modification resulted in an improved output signal.

### Evaluation: Measurements of the Second and Third Prototypes

We conducted a series of measurements of the ensemble of sensors (zipper, XYZ pad, 3 × 3 matrix) on both the second and third prototypes. The results are presented together to show how the modifications from version 2 to version 3 contribute to a significant improvement on TIZI v3.

#### Zipper

**Figure 8:**
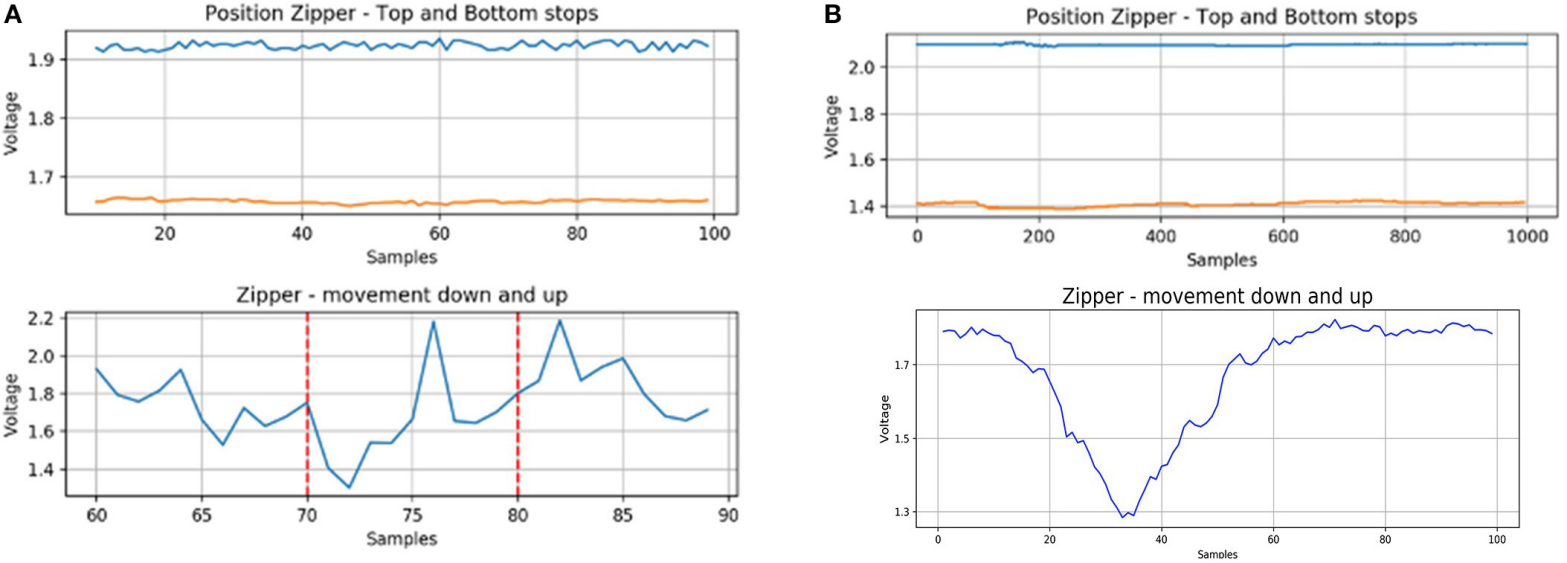
**(A)** Zipper second version; **(B)** Zipper third version. The top graphs show the reference signal from when the metal slider of the zipper is at the top (blue) and bottom (orange) stops. The bottom graphs show the zipper being moved down and up again.

The zipper works as a simple potentiometer (Figure 8). By measuring its response, we observe a clear change in the signal when the metal stop is at the bottom top or at the top stop. Likewise, we easily identify the movement of the slider moving up and down. From the second version (Figure 8A) to the third one (Figure 8B), we were able to obtain a less noisy signal with a larger amplitude (from 1.6–2 V to 1.4–2.1 V).

#### The 3 × 3 Matrix

**Figure 9:**
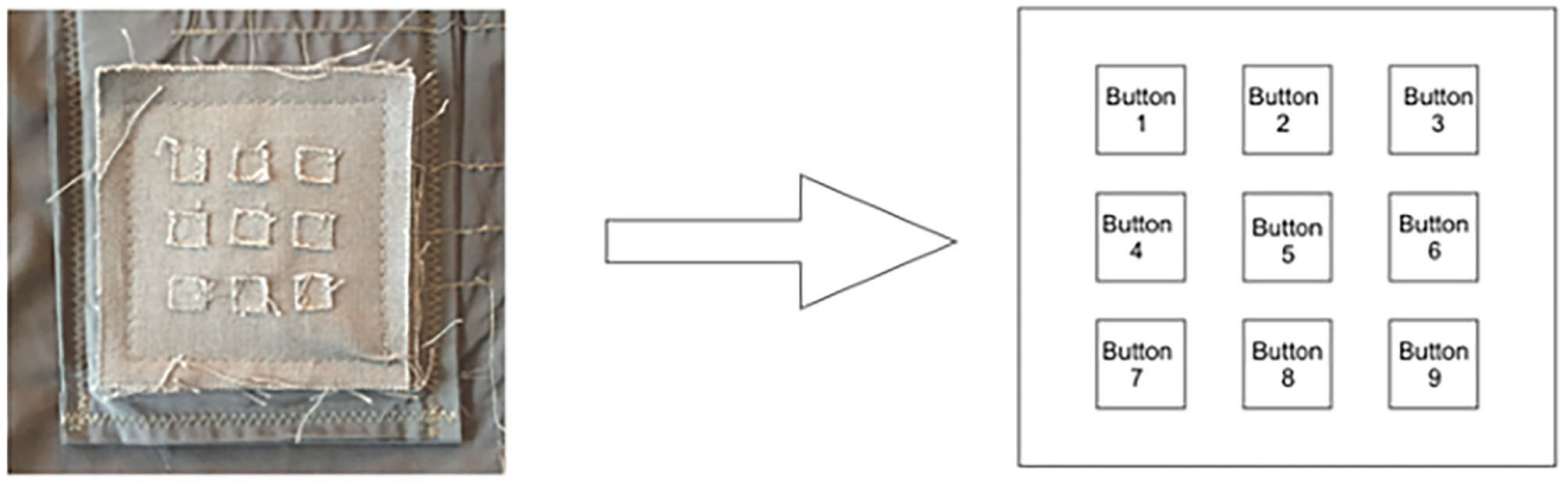
Schematic of the matrix with the mapping of the buttons; left: picture of the matrix sewn into the second version of the vest; right: the distribution of buttons in the matrix.

The 3 × 3 matrix is a touch sensor with a total of 9 buttons that can be used as a multitouch platform (Figure 9). The output signal of the matrix is continuous (we can measure pressure), but we can create a discrete on/off behavior if desired.

**Figure 10:**
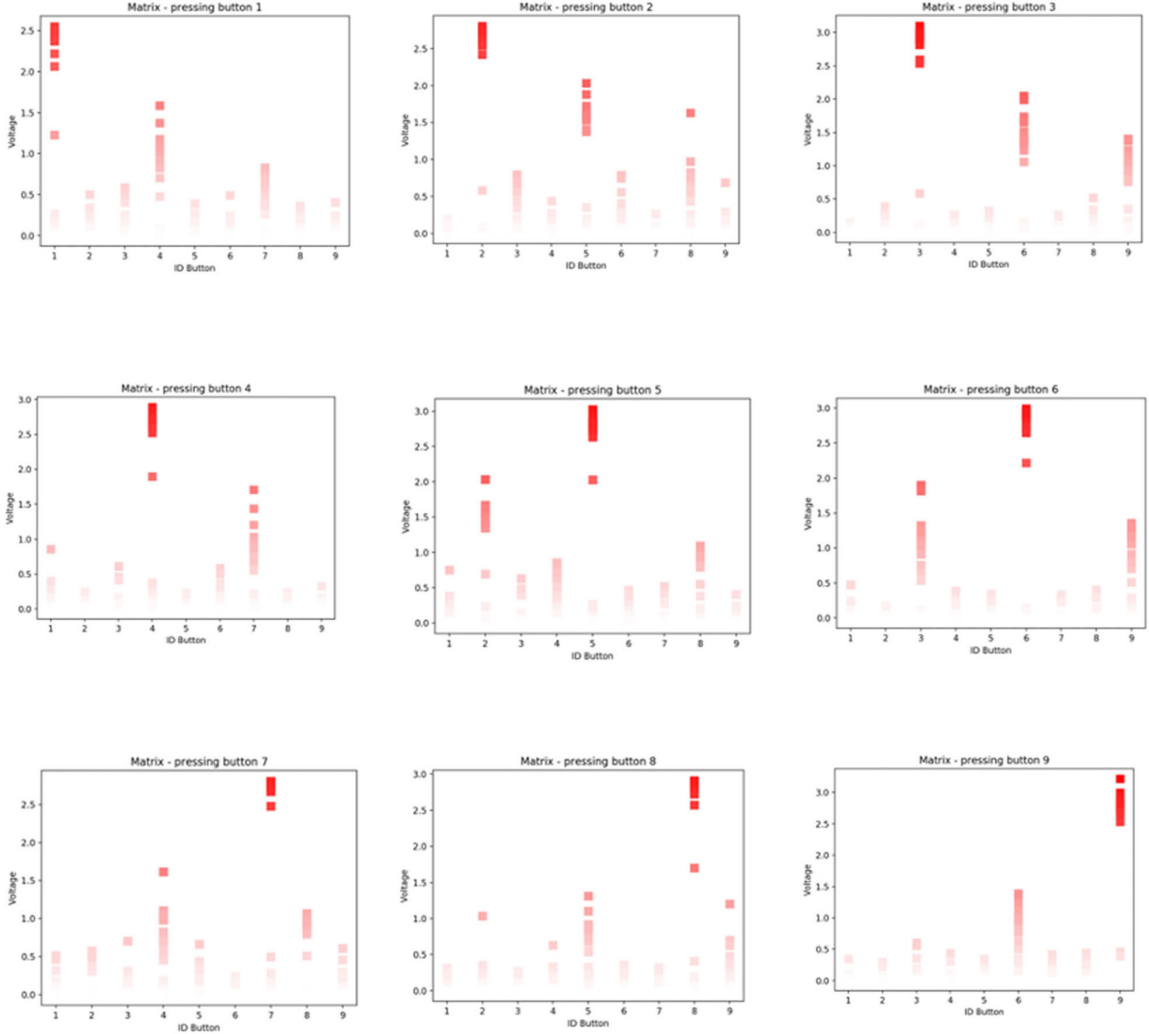
Readings of individual buttons on the matrix.

We conducted a series of simulations to observe the response of the 3 × 3 matrix when pressing the different buttons one after the other, in the order displayed in Figure 9. Figure 10 shows the results of this experiment, each graph shows the response of the matrix when pressing one button at a time. The intensity of red directly correlates with the amplitude of the signal. The bolder the red, the higher the voltage—a bold red indicates the button that was touched. The color of the trace is bright red when the amplitude of the signal is above 2V. We can observe that the button that was pressed stands out from the others. However, the buttons located in the same matrix column also detect a level of signal that could induce false positive. For example, when the user presses button 1 (top left), the other signals with higher amplitude are, in order of importance, button 4 and button 7, located below button 1 (Figure 10, top left chart).

**Figure 11:**
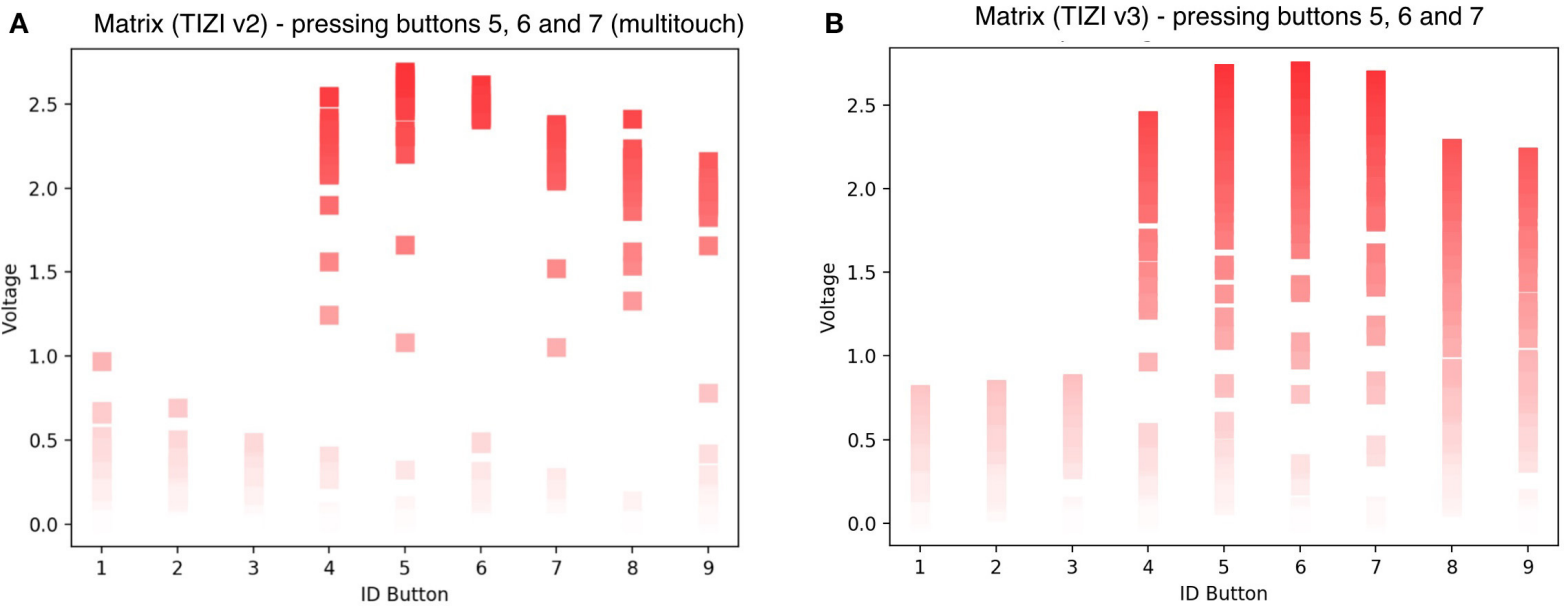
Readings of the matrix with multitouch example; **(A)** TIZI v2 vs. **(B)** TIZI v3.

Figure 11 displays the response of the 3 × 3 matrix when touching two or more buttons simultaneously. The left graph (A) shows three buttons pressed at the same time (buttons 5, 6, and 7)—we can isolate two of these buttons, but button 4 has higher amplitude than button 7, resulting in a false positive. The proximity of the buttons creates coupling between neighbors. The third version improves this (B), isolating buttons 5, 6 and 7. While we can identify which buttons are touched, other buttons detect a lot of signal that would likely result in false positives for buttons that are not touched (A). This might result from the small size of the squares (0.25 cm^2^ on the second version) and the finger therefore touching other buttons. In the third version of the vest, an increased button size (1 cm^2^) shows an improvement (B).

## Discussion

Considering our main research question (RQ1), the ideal wearable DMI that Black described during his interview mirrors Mazzola and Cherlin's (2008) emphasis on the importance of gestures in the making of improvised performances. For instance, Black associated the movements of *press, slide* and *move* as musical gestures. Also, he insisted on the importance of low latency between his gestures and the sensors' responses. Meanwhile, his concerns stressed the challenges of stage performance and international touring in terms of sensor accuracy, design robustness, and wearable hygiene.

According to our observations at the first performance test with Black, the initial TIZI prototype met his gestural and latency requirements. However, this performance test brought the team to abandon the design of the XYZ pad because of the physical discomfort and restricted movements due to the cardboard pockets underneath the pad, and because of the difficulty to find a solution to improve the reading accuracy of the X and Y axes without using a stiff surface underneath the pad. The team of designers chose to focus on improving the accuracy of the 3 × 3 multitouch matrix with pressure sensing that could suffice to meet Black's criteria to be able to *press, slide* and *move* on the wearable with several fingers on a single sensor. While Schmeder and Freed (2010) propose a simpler approach to our first attempt for an XYZ pad with accurate readings for the X and Y axes, they also use a stiff surface underneath the pad, a wood surface in their case. We plan on adapting their XYZ pad approach into our next design for a wireless feminine version. We will find a placement for this sensor that can use a stiff surface underneath the pad without creating discomfort when performing.

While the first TIZI prototype met Black's gestural and latency requirements, it did not pass the accuracy and robustness tests. The laboratory measurements of the second prototype's sensor responses showed satisfying results for the zipper as potentiometer but not for the 3 × 3 multitouch matrix with pressure sensing. Eventually, the laboratory measurements of the third prototype's matrix proved that the designer team achieved our accuracy goals.

At the end of the project, we used the Guthman Musical Instrument Competition as an opportunity to test the third prototype's basic robustness through a series of rehearsals and a stage performance with music student Rook. All the sensors worked as intended during the performance. Moreover, the device was intact after the event. While the project outcomes overall validate our approach to design a custom-made wearable that meets a world-class improviser's criteria, further research needs to be done to evaluate the hygiene and durability of a design that could be repaired by sewing damaged connections with conductive thread.

Considering our second research question (RQ2), from Black's performance test of the first TIZI prototype, we observed that the interface indeed facilitated his gestures when playing on drums and electronics simultaneously. Also, we noticed that he interacted with the wearable through innovative gestures that were not initially intended, e.g., hitting the pad with a drumstick to trigger sounds or generating a cluster by pressing his arm on the pad. In an ideal world, we would have evaluated the third prototype in touring conditions with Black. His touring schedule and our funding limitations did not allow this to happen. We intend to carry out a longitudinal performance study in the future with a male drummer or percussionist who would regularly perform with the device to investigate the full creative potential of this wearable DMI.

Considering our final research question (RQ3), the Grupp's selection as semi-finalist in the Guthman Competition enabled the team to assess the potential of transferring our custom-made wearable to another male performer. Music student Rook, who had never simultaneously performed on acoustic drums and electronics before enjoyed his experience rehearsing and performing on stage with the novel interface. This event confirms that TIZI, initially designed to meet Black's criteria, could effectively be adopted by a less-accomplished male improviser. While Black aimed to incorporate the wearable into his already-defined concept of acoustic drums with electronics performance, Rook composed the competition demonstration with the goal of highlighting the functionalities of the device. This situation led Rook to map his gestures with sound effects completely differently than Black used during the CIRMMT performance test with the initial prototype. According to Grupp, the unlimited personalisation and creative options that TIZI offers, i.e., leaving the mapping to the discretion of its user, was key to Rook's pleasure when experimenting with the interface.

## Conclusion and Future Research

Our case study presents the technical details of three prototypes of a wearable DMI that were tested in real-life scenarios by two improvisers of different levels of expertise. Throughout the study, the improvisers were free to map the sensors to the sounds and effects of their choice.

Outcomes of a range of evaluation methods validate our pre-established approach of drawing upon enactive knowledge to augment an everyday well-known object as the basis of the gestural interface for improvised performances. Furthermore, the fact that this everyday object is wearable eases the embodiment of the gestural interface. By solving initial technical issues based on a performance test with a world-class drummer, we could develop a new technique to integrate several sensors that are wired by conductive threads on a garment subject to intense movements and sweat. We also contributed to technological advancement with the development of a fabric 3 × 3 matrix that enables multitouch functions and pressure sensing. With the goal of creating a gestural interface that would be generic enough to be adopted by other male drummers or percussionists, we developed sensors that are modular and interchangeable. While this customizable design still needs improvements and tests before we can call it marketable, these first results encourage us to pursue our approach for future research.

Our research contributes to the Performance Science and DMI communities by showing the benefit and constraints of involving music performers in research projects. We suggest that the design trajectory presented in this case study can provide DMI designers with a novel approach to define criteria and framework by establishing initial goals from an interview with the performer, and to explore different methods to evaluate the prototypes. Moreover, our approach showed that bringing a world-class improviser to the center of the design process can help designers reduce complexity and improve virtuosic features when conceptualizing a new DMI. Previous research in improvisation studies highlights that if gestures are facilitated and mastered, the musician can focus on the performance process and outcome (Pras, 2016). Similarly, if the musician is an active participant of the design process, the designer can focus on the technical steps required to create an interface that is open, intuitive, and relevant for musicians of different level of expertise. With this in mind, the collective and multidisciplinary process described in this paper can speed up the experimentation stage of DMI design. We believe that the inclusion of a world-class improviser at the center of the design process may generate more interest in DMI laboratories from musicians outside of academia and dedicated niche conferences. Also, high-level touring musicians may be more inclined to challenge the DMI community by exposing their wishes, constraints, and requirements.

Our future plans include a design residency with Diana Policarpo^13^, a visual artist and free improviser based in Lisbon and London whose work merges visual and musical media, including drawing, sculpture, large-scale performances, and multi-channel sound installation. Policarpo was chosen because we knew that her improvisation approach and performance gestures would differ from Black's. For instance, she uses sounds as a plastic material for both her art installations and electroacoustic performances. She is specifically interested in how the room affects the generated sound, and in creating unpredictable scenarios that trigger specific gestural responses. Also, she has an interest for DMI design and she previously tried creating a portable drum attached to her torso as a wearable garment^14^.

In October 2017, Rodrigues interviewed Policarpo via Skype. During this interview, Rodrigues explained the main features of the third TIZI prototype and asked a few questions about her performance gestures, as well as about the sensors and mapping preferences that she would like to use or change. Policarpo pictures a whole suit rather than a vest, to be able to use her complete body instead of her torso only. The garment should be wireless as she would like to be able to walk around the room when performing. During her interview, she provided several references from female artists' works to help imagine her dream wearable, such as Laurie Anderson's bodysuit (Anderson, 1986, p. 8–45), Letitia Sonami's glove (Sonami, 2014, p. 21–56), and Pamela Z's bracelet (Z, 2017). These examples reveal her interest in having some type of device in her hands as well. This approach differs from Black's who preferred to have his arms and hands completely free for his drumming gestures. Policarpo sees the gestural interface as a creative partner where the garment can and should inspire her to respond. She thus extends Black's vision of an electronic device that he can fully control. Ideally, she would like to be able to enter into a dialogue with the interface. We thus envisage the introduction of haptic feedback, following the example of the Whole-body Haptic Suit (Giordano et al., 2015; Lamontagne et al., 2015). Finally, Policarpo also shows interest in having a system that would trigger percussive sounds by tapping her body. We need a more thorough assessment of the gestures she uses in her performances to choose which sensors will best respond to her needs, and to adapt the technology created in TIZI to a version of the interface suited to the female body. This interview also enables us to imagine a female body version of the wearable without trying to replicate the style of TIZI. We plan on exploring new sensors for her bodysuit, e.g., haptic feedback sensors to respond to her desire for dialogue with the interface in solo performance. Our extended vision for this design project thus became a modular wearable interface that uses the knowledge of making and incorporating specific sensors on the garment according to specific improvised performance needs.

## Data Availability Statement

The measurement of the wearable sensor responses is available upon request to mailisr@gmail.com.

## Ethics Statement

Ethical review and approval were not required for this type of study in accordance with the local legislation and institutional requirements. This research did not include the participation of subjects but was an artistic project with four musicians who gave their oral consent to participate in the project and to be named in the paper. Jim Black and Felix del Tredici gave their written consent for the inclusion of the pictures and videos in this paper.

## Author Contributions

AP was PI of the research project, supervised VG throughout the design trajectory, brought Jim Black and Felix del Tredici on the project, coordinated the performance test with Black at CIRMMT, filmed and edited a video of this performance test, and wrote 40-50% of the paper. MR was hired as a post-doctorate researcher on the project by Wanderley to mentor VG for the creation of the interface, performed all the sensor measurements, brought Diana Policarpo on the project, and wrote 40-50% of the paper. VG received a student grant to design the wearable during her residency at IDMIL, McGill, completed the third prototype on her own after she had left IDMIL, produced the video demo of the interface, brought Blake Rook on the project, and presented her design with Rook at the 2017 International Guthman Musical Instrument Competition. MW hosted VG and hired MR for the project at IDMIL for two months, brought his technological expertise at meetings during the design process, introduced the team to Rodolphe Koehly, and wrote 10-20% of the paper. All authors contributed to the article and approved the submitted version.

## Conflict of Interest

The authors declare that the research was conducted in the absence of any commercial or financial relationships that could be construed as a potential conflict of interest.

## Footnotes

^1^This session was conducted with video anthropologist Grégoire Lavergne to study improvisation practices with electronic devices in New York (Lavergne and Pras, 2014). A video excerpt of this session is discussed in Jim Black's portrait *L'Improvibatteur* by linguists Cloiseau and Cance (2016).

^2^At least in New York, *free jazz* has a negative connotation among certain improvisers' communities (Pras, 2016).

^3^See two videos of Satoshi's solo performance on percussions and electronics: one at NYU published in Pras (2016) and Dew Drops as part of the live@CIRMMT event named Free Drums, organized by Pras and Rodrigues in Nov 2015.

^4^A community around Arduino (www.arduino.org) regularly exchanges knowledge and discusses projects, leading other tech companies to develop non-expensive micro-controllers, e.g., Intel, Adafruit, that are compatible with Arduino and may be programmed with the same Integrated Development Environment (IDE).

^5^The international conference on new instruments for musical expression: www.nime.org.

^6^The Portrait of a Lady: Video Improvisations with Jim Black performance at Stetson University on February 11, 2016, featured improvisations of Black on drums and electronics, and live video by Matt Roberts' students including Grupp. See the excerpt from 31:00 to the end during which Black uses the ROLI Seaboard RISE 25 to play a bass line when simultaneously performing on acoustic drums.

^7^Rodolphe Koehly founded a company (TreeCell4U) that designs electronic sensors using paper and other non-polymerous materials (Koehly, 2011).

^8^These pieces include *40 Icons on Art and Music* for trombone, narrator and bodysuitbelt; *Fragile Dis equilibria* for trombone and bodysuitscore; *Fragile Disequilibria 2.0* for trombone, electronics, and bodysuitscore; *TWINE* for trombone, cello, bass clarinet, and body suits; and *Niemandslandhymnen* for singers and bodysuitbelts (see West West et al., 2019; Turchet et al., 2020).

^9^McGill jazz faculty professor John Hollenbeck provided the drum kit.

^10^Velostat. (2019). Retrieved from: https://en.wikipedia.org/wiki/Velostat (accessed June 14, 2020).

^11^Available online at: https://www.adafruit.com/product/659.

^12^A video montage of the duo performance with trombonist Del Tredici is available in Supplementary Materials. A version with high quality is available on Vimeo.

^13^Policarpo has performed around the world and her sound installations have been shown in both solo and group exhibitions around Europe and North America.

^14^Breastplate—marching drum part of the live performance *Here on Earth as Well*, London 2013.
